# Assessing Genetic Diversity in Endangered Plant *Orchidantha chinensis*: Chloroplast Genome Assembly and Simple Sequence Repeat Marker-Based Evaluation

**DOI:** 10.3390/ijms252011137

**Published:** 2024-10-17

**Authors:** Yiwei Zhou, Jianjun Tan, Lishan Huang, Yuanjun Ye, Yechun Xu

**Affiliations:** 1 Environmental Horticulture Research Institute, Guangdong Academy of Agricultural Sciences, Guangzhou 510640, China; zhouyiwei6333@163.com (Y.Z.);; 2 Guangdong Key Laboratory of Ornamental Plant Germplasm Innovation and Utilization, Guangzhou 510640, China

**Keywords:** *Orchidantha chinensis*, population structure, cpSSR, EST-SSR, chloroplast genome

## Abstract

*Orchidantha chinensis* T. L. Wu, an endemic species in China, is listed as a key protected wild plant in Guangdong Province. However, the lack of reports on the chloroplast genome and simple sequence repeat (SSR) markers has hindered the assessment of its genetic diversity and conservation strategies. The limited number of molecular markers to assess the genetic diversity of this species, and thus develop proper conservation strategies, highlighted the urgent need to develop new ones. This study developed new SSR markers and investigated genetic variation using 96 samples of *O. chinensis* from seven populations. Through high-throughput sequencing, a complete chloroplast genome of 134,407 bp was assembled. A maximum-likelihood phylogenetic tree, based on the chloroplast genome, showed that *O. chinensis* is closely related to *Ravenala madagascariensis*. The study identified 52 chloroplast SSRs (cpSSRs) and 5094 expressed sequence tag SSRs (EST-SSRs) loci from the chloroplast genome and leaf transcriptome, respectively. Twenty-one polymorphic SSRs (seven cpSSRs and fourteen EST-SSRs) were selected to evaluate the genetic variation in 96 accessions across seven populations. Among these markers, one cpSSR and 11 EST-SSRs had high polymorphism information content (>0.5). Cluster, principal coordinate, and genetic structure analyses indicated that groups G1 and G6 were distinct from the other five groups. However, an analysis of molecular variance showed greater variation within groups than among groups. The genetic distance among the populations was significantly positively correlated with geographical distance. These findings provide new markers for studying the genetic variability of *O. chinensis* and offer a theoretical foundation for its conservation strategies.

## 1. Introduction

The genus *Orchidantha* N. E. Brown (family Lowiaceae) is the sole genus within the order Zingiberales. Currently, at least 27 species have been identified worldwide, with recent discoveries including *O. sarawakensis* Syauqina & Meekiong [1], *O. crassinervia* P. Zou & X. A. Cai [2], *O. virosa* Škorničk. & Q. B. Nguyen [3], *O. stercorea* H. Ð. Tran & Škorničk. [4], and *O. yunnanensis* P. Zou, C. F. Xiao & Škorničk. [5]. Lowiaceae is believed to have originated in northeastern Sundaland, from where it spread to Indochina, the Thai-Malay Peninsula, and Borneo [6]. This family, along with Musaceae, Heliconiaceae, and Strelitziaceae, is considered part of the “banana group” within Zingiberales, characterized by having five to six fertile stamens [7]. In 2005, Johansen’s research identified Lowiaceae as the sister group to all other families within Zingiberales, with the Bornean group forming a strongly supported monophyletic clade [6]. Subsequent genetic analyses have positioned Lowiaceae as the sister group to Strelitziaceae [8,9,10,11]. Beyond genetic data, similarities in inflorescence structure and floral development between Lowiaceae and Strelitziaceae further support their sister relationship. In a phylogenetic analysis of 17 Lowiaceae samples, the position of *O. chinensis* from mainland China was not supported [6], indicating an unclear evolutionary position within Lowiaceae. Further research is needed to clarify its evolutionary status.

*Orchidantha chinensis*, endemic to China, is found only in the Guangdong Province and Guangxi Zhuang Autonomous Region. It is listed in the Red List of Chinese Biodiversity (https://www.iplant.cn/rep/prot/Orchidantha%20chinensis (accessed on 6 September 2024)) and was also designated as a key protected wild plant by Guangdong Province in 2023 (https://www.gd.gov.cn/xxts/content/post_4142096.html (accessed on 6 September 2024)). Additionally, *O. chinensis* has medicinal properties [12,13]. Based on our years of field surveys and monitoring, *O. chinensis* primarily reproduces asexually through underground rhizomes. However, it can also produce seeds naturally, although these seeds are highly susceptible to predation by wildlife. Previous studies have focused mainly on its anatomy [14,15] and cytology [16,17], with limited reports on genetic diversity and population biology. Li Rong et al. used 10 ISSR markers to analyze the genetic diversity of seven *O. chinensis* populations, followed by an analysis of five leaf morphological traits and a preliminary investigation into the possible causes of its endangerment [18,19]. However, the development of molecular markers based on high-throughput sequencing has not been reported, hindering further research into the endangerment mechanisms and conservation strategies for *O. chinensis*.

Simple sequence repeat (SSR) markers, also known as microsatellite markers, are widely used in studies of plant genetic diversity and population genetics due to their high polymorphism and reproducibility [20,21]. Chloroplast SSRs (cpSSRs), typically found in the non-coding regions of the chloroplast genome, often show intraspecific variation in the repeat number [22,23]. Due to their uniparental inheritance, chloroplast markers can be more effective than nuclear markers for population subdivision, thus better revealing genetic variation within and between species. Their recognized potential to complement nuclear genetic markers in population genetics and biogeography studies is well established [24,25,26]. Additionally, Expressed Sequence Tag (EST)-SSRs, due to their proximity to regulatory or coding regions, are potentially subject to selective pressures, and variations within these repetitive sequences may influence the expression patterns of downstream genes. Consequently, variations in EST-associated SSRs could be implicated in adaptive genetic variations. Given the lack of reports on cpSSRs and EST-SSRs for *O. chinensis*, developing these markers could enhance our understanding of the species’ evolution, diversity, risk of extinction, and conservation monitoring measures.

This study focuses on wild populations of *O. chinensis* in Yangchun County, Yangjiang City, Guangdong Province, China. First, we assembled the chloroplast genome of *O. chinensis* using high-throughput sequencing to evaluate its genetic relationships with other families in Zingiberales. We then developed cpSSR and EST-SSR markers suitable for *O. chinensis*, providing new tools for studying its genetic structure and population differentiation. Using these newly developed markers, we analyzed the genetic diversity of different populations and examined the relationship between genetic and geographic distances, aiding in the further investigation of the intrinsic causes of this species’ endangerment.

## 2. Results

### 2.1. Assembly, Annotation, and Phylogenetic Analysis of Chloroplast Genome of O. chinensis

**Figure 1:**
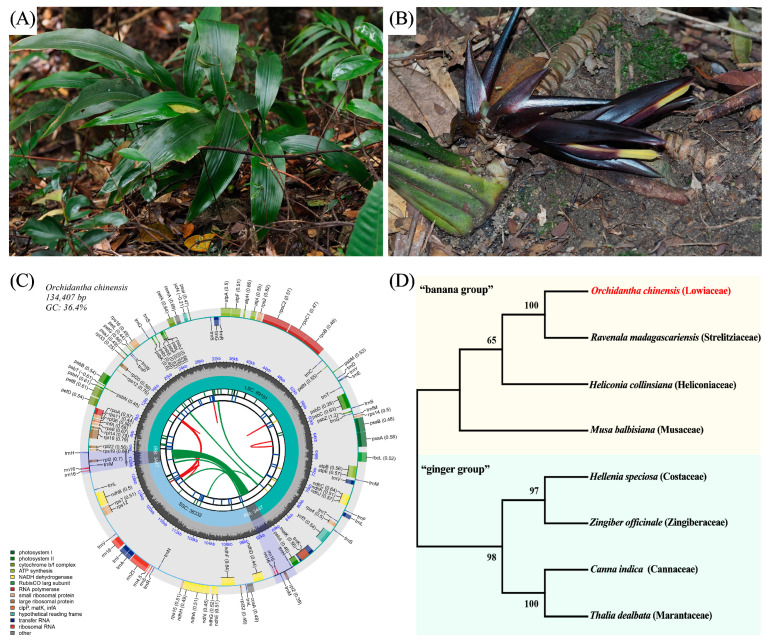
The chloroplast genome of *O. chinensis* and the phylogenetic tree of representative species from eight families in Zingiberales. (**A**) Plants of *O. chinensis*. (**B**) The inflorescence of *O. chinensis*. (**C**) The chloroplast genome map of *O. chinensis*. (**D**) The maximum-likelihood phylogeny tree obtained from eight complete chloroplast sequences in Zingiberales. The position of *O. chinensis* has been highlighted in red.

The chloroplast genome of *O. chinensis* spans 134,407 base pairs and exhibits a quadripartite structure (Figure 1). This structure includes a large single-copy (LSC) region of 89,181 base pairs, a small single-copy (SSC) region of 38,332 base pairs, and two inverted repeat (IR) regions, each being 6894 base pairs in length (Figure 1C). The genome’s overall GC content is 36.4%, which is higher than that of the LSC (38.1%) and SSC (37.8%) regions, but lower than that of the IR regions (39.58%). It encodes 108 unique genes, comprising 71 protein-coding genes (PCGs), 33 tRNAs, and four rRNAs (Table 1). Introns are present in 16 genes: 15 genes (*rpoC1*, *ndhB*, *ndhA*, *atpF*, *rps7*, *rpl16*, *petB*, *petD*, *rps16*, *trnL-UAA*, *trnG-UCC*, *trnI-GAU*, *trnA-UGC*, *trnK-UUU*, *trnV-UAC*) contain a single intron, while one gene (*ycf3*) contains two introns (Table S1). A maximum-likelihood phylogenetic analysis revealed that *O. chinensis*, *Ravenala madagascariensis*, *Heliconia collinsiana*, and *Musa balbisiana* cluster together, forming the “banana group”, while *Hellenia speciosa*, *Zingiber officinale*, *Canna indica*, and *Thalia dealbata* form the “ginger group” (Figure 1D). *O. chinensis* is most closely related to *R. madagascariensis* of the Strelitziaceae family, with strong support.

### 2.2. SSR Identification of O. chinensis and Polymorphism Detection

To develop SSR markers for *O. chinensis*, we first used the MIcroSAtellite (MISA) identification tool to identify SSR loci in the assembled chloroplast genome. A total of 52 SSR loci were identified, including 44 mononucleotide repeats, 5 dinucleotide repeats, 1 trinucleotide repeat, and 2 hexanucleotide repeats (Table S2). The majority of these SSRs (78.84%) are composed of A/T motifs.

To develop additional SSR markers, we conducted RNA sequencing on *O. chinensis* to develop EST-SSR markers. RNA sequencing of *O. chinensis* leaves yielded 14,034 unigenes with a total length of 27,803,090 bp after stringent quality control and assembly (Table S3). Using the MISA tool, 5094 SSR loci were identified, and distributed across 3877 unigenes, with an SSR occurrence rate of 27.63%. Among these, 918 unigenes contained more than one SSR locus (23.68%), and 309 unigenes contained compound SSR loci (7.97%) (Table S3). A further analysis revealed 70 different repeat motifs, including 2 mononucleotide, 4 dinucleotide, 10 trinucleotide, 10 tetranucleotide, 9 pentanucleotide, and 26 hexanucleotide motifs (Table S4). Mononucleotide repeats were the most abundant (2177; 42.74%), followed by trinucleotide (1701; 33.39%) and dinucleotide repeats (1107; 21.73%). Tetranucleotide, pentanucleotide, and hexanucleotide repeats were less common, with 58, 18, and 33 repeats, respectively (Table S5). The average distribution distance was greatest for pentanucleotide repeats (1544.62 kb) and shortest for mononucleotide repeats (12.77 kb) (Table S5). SSR repeat numbers ranged from 5 to 34, with the majority (80.29%) falling within the 5–11 range (Figure S1A; Table S6). SSR loci with repeat numbers of 10, 5, 6, and 11 were the most common, accounting for 20.04%, 18.73%, 14.49%, and 10.60% of the total SSRs, respectively (Figure S1A; Table S6). SSR loci with more than 15 repeats were rare, comprising only 4.71%. Significant differences were observed in the composition of different SSR types (Figure S1B). Among mononucleotide repeats, A/T motifs were predominant (98.9%). AG/CT was the most common dinucleotide repeat (64.00%), AGG/CCT was the most frequent trinucleotide repeat (31.10%), AAAG/CTTT was the most abundant tetranucleotide repeat (24.1%), AGAAG/CCTCT was the most common pentanucleotide repeat (33.3%), and AGGCGG/CCGCCT was the most prevalent hexanucleotide repeat (12.1%). A comprehensive analysis of all motif types revealed that A/T repeats were the most numerous (2154; 42.29%), followed by AG/CT, AGG/CCT, AAG/CTT, AT/AT, CCG/CGG, AGC/CTG, and AC/GT, with counts of 708, 529, 300, 296, 263, 217, 102, and 100, respectively. The remaining 62 motifs each had fewer than 100 repeats (Figure S1C).

After preliminary screening, 7 cpSSR and 14 EST-SSR polymorphic primers were selected for a further analysis, all exhibiting a good polymorphism and reproducibility. The seven cpSSRs included two compound SSRs, two hexanucleotides, and three mononucleotides (Table S7). Their Major Allele Frequency ranged from 0.52 to 0.98, with a mean of 0.79. The number of alleles (*Na*) ranged from 2 to 5, with a mean of 3.14. Gene diversity ranged from 0.04 to 0.59, with a mean of 0.32. Polymorphism information content (PIC) values ranged from 0.04 to 0.52, with a mean of 0.29. OccpSSR45 had the highest gene diversity and *PIC* values (Table 2). The 14 EST-SSRs included 13 trinucleotide repeats and 1 tetranucleotide repeat (Table S8). Their Major Allele Frequency ranged from 0.20 to 0.80, with a mean of 0.39; *Na* ranged from 3 to 29, with a mean of 9.93; gene diversity ranged from 0.33 to 0.89, with a mean of 0.73; and PIC values ranged from 0.31 to 0.89, with a mean of 0.69 (Table 2). These results indicate that the selected SSR markers exhibit a good polymorphism. The Major Allele Frequency of the seven populations ranged from 0.59 to 0.97, with a mean of 0.76; *Na* ranged from 1.29 to 3.43, with a mean of 2.50; gene diversity ranged from 0.05 to 0.50, with a mean of 0.32; and *PIC* values ranged from 0.05 to 0.46, with a mean of 0.29 (Table 3). G1 had the highest *PIC* value, while G5 had the lowest.

### 2.3. Genetic Diversity and Population Structure Analysis

**Figure 2:**
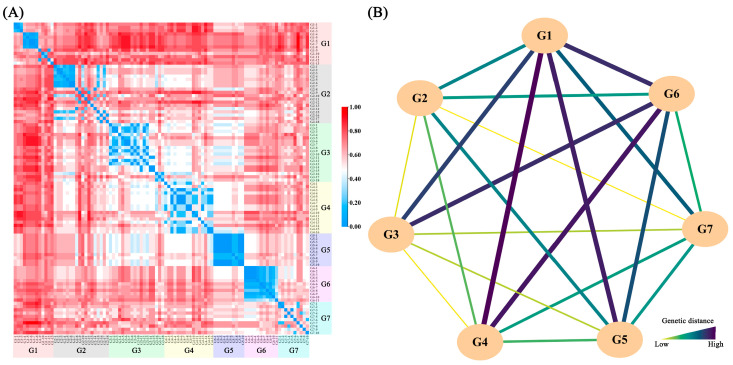
Genetic distance between different individuals (**A**) and populations (**B**) of *O. chinensis*.

The heatmap of genetic distances among the 96 samples (Figure 2A) shows that many accessions from different populations have small genetic distances (Table S9). The genetic distance among the seven populations ranged from 0.34 to 0.57, with the greatest distance between G1 and G4 and the smallest between G2 and G3. Interestingly, G1 and G6 had genetic distances greater than 0.4 from other populations, indicating significant genetic differentiation (Figure 2B; Table S10). A Neighbor-Joining (NJ) clustering analysis of the 96 samples revealed that most samples from the same population clustered together, with only a few samples from different populations intermixing (Figure S2A). NJ clustering of the seven populations showed that G3, G4, and G5 formed a small clade, G6 and G1 clustered together, and G2 and G7 clustered together, indicating greater genetic similarity (Figure S2B).

**Figure 3:**
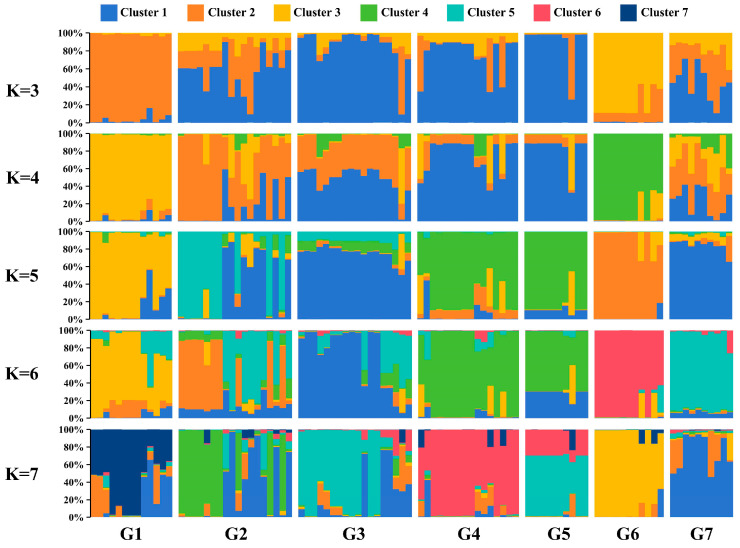
Population structure analysis of 96 *O. chinensis* accessions across seven groups.

A Principal Coordinates Analysis (PCoA) of the 96 accessions showed that the first three axes explained 13.20%, 10.40%, and 9.09% of the variation, respectively. G1 and G6 accessions were relatively distant from other groups, while G2 and G7 were closer, and G3, G4, and G5 were relatively close, consistent with the NJ tree results. PCoA of the seven populations further confirmed this pattern (Figure S2C,D). A population genetic structure analysis showed that the optimal K value was 7, with the largest ΔK value (Table S11; Figure S3). However, other potential clusters were observed at K = 4 and 5. The LnP(K) value increased with larger K values (Figure S4). The visualization of the sample Q values for these K values revealed that G1 and G6 were distinct from the other five groups, while G2, G3, and G4 were more similar (Figure 3). Specifically, when K = 3, G1 and G6 formed two distinct clusters, while G2, G3, G4, G5, and G7 appeared to originate from a common ancestor. When K = 4, G1 and G6 remained separated; G3, G4, and G5 were more closely related; and G2 and G7 were relatively mixed. At K = 5 and 6, G1, G6, and G7 exhibited three distinct clusters, and G2 and G3 showed some separation but also heterogeneity, while G4 and G5 originated from a common ancestor. At K = 7, G1, G4, G6, and G7 displayed four distinct clusters, while G2, G3, and G5 showed heterogeneity. G2 and G3 contained genetic information from G7, and G5 contained genetic information from G3 and G4.

### 2.4. AMOVA and Correlation Analysis of Genetic and Geographic Distances

**Figure 4:**
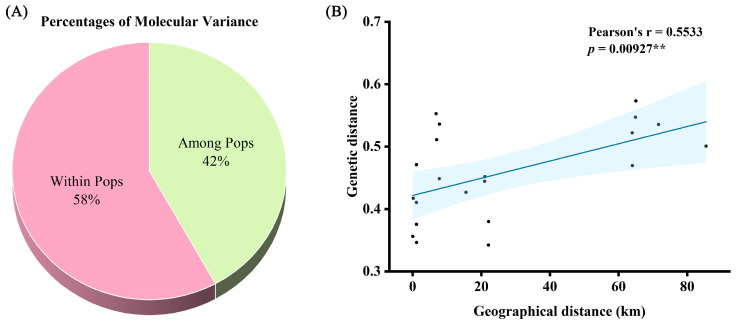
AMOVA analysis and general linear regression analysis between genetic distance and geographical distance. (**A**) Percentages of molecular variance. (**B**) General linear regression analysis. “**” indicates *p* < 0.01.

Analysis of molecular variance (AMOVA) results showed that differences among the seven populations were small (42%), with most genetic variation occurring within populations (58%) (Figure 4A; Table 4). To further analyze whether geographic distance affects genetic differentiation in *O. chinensis* populations, a general linear regression analysis was performed on genetic and geographic distances among the seven populations. The results showed a Pearson’s r of 0.5533 and a *p*-value of 0.00927 (<0.01), indicating a highly significant positive correlation between geographic and genetic distances (Figure 4B).

## 3. Discussion

This study represents the first assembly of the chloroplast genome of *O. chinensis*, providing crucial bioinformatic resources for the genetic diversity research of this species. The inferred phylogenetic tree indicates that *O. chinensis* (Lowiaceae) is closely related to *Ravenala madagascariensis* (Strelitziaceae), corroborating previous findings based on other molecular markers and floral morphology that suggest a closer relationship between Lowiaceae and Heliconiaceae [7,11]. Interestingly, previous analyses of genetic differences within the genus *Orchidantha* have not clarified the exact relationship between *O. chinensis* and other *Orchidantha* species distributed in Southeast Asia [6,7]. Therefore, it is imperative to analyze the chloroplast genomes of other *Orchidantha* species to resolve genetic differentiation within the genus.

To date, researchers have primarily focused on the formation and distribution of *Orchidantha* species in Southeast Asia, with little attention given to those endemic to China. Although genetic diversity studies using ISSR markers for *O. chinensis* have been reported [18], SSR molecular markers offer superior advantages in terms of reproducibility, applicability, and specificity [27]. Furthermore, SSR markers exhibit greater specificity, enabling a more precise identification of genetic variations. These attributes make SSR markers a more robust tool for studying the genetic diversity and population structure of *O. chinensis*. To elucidate the genetic differences among seven wild populations of *O. chinensis*, we developed 7 cpSSR and 14 EST-SSR polymorphic primers through high-throughput sequencing in this study. The *PIC* values for cpSSR markers ranged from 0.04 to 0.52, while those for EST-SSR ranged from 0.31 to 0.89, indicating a higher polymorphism for EST-SSR. Generally, a PIC value greater than 0.5 indicates a high polymorphism, a value between 0.25 and 0.5 indicates a moderate polymorphism, and a value less than 0.25 indicates a low polymorphism [28]. In this study, 1 cpSSR and 11 EST-SSR markers had *PIC* values above 0.5, providing highly polymorphic markers for studying the genetic diversity and species formation of *O. chinensis*. Additionally, we analyzed the genetic statistics among the seven populations, finding that G1 had the highest *Na* and *PIC* values. Five populations (G1, G2, G3, G4, G7) had *PIC* values between 0.25 and 0.5, while populations G5 and G7 had *PIC* values below 0.25. This indicates varying degrees of genetic variation within populations from different locations, which may be related to the environmental conditions and reproductive strategies of *O. chinensis*.

Genetic diversity and structure analyses of seven different populations revealed some differences between G1 and G6 and other populations, although these differences were relatively small. There was a significant positive correlation between geographic and genetic distances. Since G1 is relatively distant from other populations, its genetic distances are larger, suggesting that geographic separation limits gene flow between populations. Additionally, G2, G3, G4, and G5 are all located on Gou Ji Ding Mountain in Yangchun County, Guangdong Province, China, and are geographically close to each other. Genetic structure analysis results at K = 3 support that they originate from the same ancestor, and their altitudes are higher compared to other populations. Notably, although G7 is geographically more than 20 km away from G2, G3, G4, and G5, it still shares some genetic information with them, suggesting possible gene flow through some medium. Therefore, in situ conservation should be prioritized for *O. chinensis*, and ex situ conservation should aim to preserve plants from different populations. The AMOVA analysis of the seven populations indicated that genetic differentiation within populations was significantly higher than between populations, consistent with previous studies in the western Tianshan Mountains [29]. The high genetic variation within populations suggests that *O. chinensis* primarily reproduces through cross-pollination, as most genetic variation in cross-pollinated plants is distributed among individuals within populations [30]. Previous studies have shown that *O. chinensis* attracts scarabaeid dung beetles from the genus *Onthophagus Latreille* for pollination by emitting a scent similar to rotting meat [31]. This suggests that *O. chinensis* primarily forms seeds through cross-pollination, which likely facilitates gene flow between different populations or the emergence of new populations. However, further observation and experimental evidence are needed.

Maintaining genetic variation in wild populations is crucial for the conservation of endangered plants [29,32,33]. Due to its low reproductive coefficient and medicinal value, *O. chinensis* is currently on the brink of extinction due to illegal harvesting. Curiously, *O. chinensis* can naturally produce a large number of seeds in the wild, but large populations have not been observed. Interestingly, recent field observations revealed that most seeds are eaten by rodents when the fruit matures, suggesting that small, closely distributed populations may be formed through rodent-mediated seed dispersal. Rodent seed predation is not uncommon, as seen in species like *Pinus sylvestris*, *Quercus robur*, and *Fagus sylvatica* [34]. However, due to the difficulty in propagating *O. chinensis*, extensive seed predation by rodents severely impacts its natural reproduction. The reason why rodents particularly favor *O. chinensis* seeds remains a mystery. Future research will focus on the role of these animals in influencing the migration and distribution of *O. chinensis* populations, especially using the polymorphic markers developed in this study. To protect this endangered plant, *O. chinensis* has been listed as a nationally and provincially protected species in recent years. Preliminary tissue culture has produced aseptic seedlings of *O. chinensis*, but they grow much slower than typical plant tissue culture seedlings. Overall, saving this endangered species remains challenging.

## 4. Materials and Methods

### 4.1. Plant Materials

Leaf samples from 96 individuals across seven wild populations of *O. chinensis* were collected in Yangchun County, Yangjiang City, Guangdong Province, China. The collection sites for the seven populations were G1 (E 111°26′53″, N 21°53′47″), G2 (E 111°35′58″, N 22°10′43″), G3 (E 111°35′55″, N 22°10′41″), G4 (E 111°36′10″, N 22°11′30″), G5 (E 111°36′26″, N 22°11′17″), G6 (E 111°35′26″, N 22°17′41″), and G7 (E 111°44′45″, N 22°28′52″), with 13, 18, 18, 16, 10, 11, and 10 plants, respectively. The leaves were flash-frozen in liquid nitrogen and stored in liquid nitrogen tanks. DNA and RNA extraction from the leaves was performed by Biomarker Technologies Co., Ltd., Beijing, China, followed by whole-genome resequencing and transcriptome sequencing on the Illumina platform.

### 4.2. Assembly of the O. chinensis Chloroplast Genome

Using the CTAB method, DNA was extracted from fresh leaves. A Nanodrop ND-1000 spectrophotometer (Thermo Scientific Inc., Waltham, MA, USA) was used to measure the DNA concentration. After quality control, the genomic DNA was fragmented using Covaris ultrasonicators (Covaris, Woburn, MA, USA), and the fragments were purified, end-repaired, A-tailed, and ligated to sequencing adapters. The fragments were size-selected using agarose gel electrophoresis, PCR-amplified to form sequencing libraries, and sequenced (paired-end) on the Illumina platform. The library preparation kit used was the NEBNext^®^ Ultra™ RNA Library Prep Kit (Illumina, San Diego, CA, USA). De novo assembly of the chloroplast genome was performed using GetOrganelle v1.7.2 [35] with default settings (-R 15; -k 21, 45, 65, 85, 105; -F embplant_pt). The assembled chloroplast genome was annotated using PGA [36] and manually corrected for start and stop codons. These annotated regions include the large single-copy (LSC) region, small single-copy (SSC) region, and inverted repeat (IR) region. Additionally, the annotated genes were categorized into three main groups based on previous studies: genes for photosynthesis, self-replication, and other genes [37]. The final genome map of *O. chinensis* was generated using CPGview (http://www.1kmpg.cn/cpgview (accessed on 15 July 2024)) [38]. The complete chloroplast genome sequence of *O. chinensis* was uploaded to GenBank (PQ415630).

### 4.3. RNA Sequencing and Assembly of O. chinensis Leaves

Fresh leaves were used to extract total RNA, which was then assessed for quality and quantity with the Agilent Bioanalyzer (Agilent Technologies, Santa Clara, CA, USA). RNA integrity was confirmed through agarose gel electrophoresis. For RNA sequencing, cDNA libraries were prepared using the Illumina Hicseq™ RNA sample preparation kit (Illumina, San Diego, CA, USA) according to the manufacturer’s instructions. The library size and concentration were measured using Qubit 2.0 and Bioanalyzer 2100 by Agilent (Agilent Technologies Inc., USA). Paired-end (150 PE) sequencing was performed on the Illumina Hi-Seq 6000 platform. Low-quality reads and adapters were removed from the raw data using Trimmomatic [39]. The clean reads were assembled de novo into contigs using Trinity program 2 [40] with an optimized k-mer length of 25 and a group pair distance of 300.

### 4.4. Identification of SSR Loci and Primer Design

Both cpSSRs and EST-SSRs were identified in the chloroplast genome or unigene sequences using the MIcroSAtellite identification tool (MISA, https://webblast.ipk-gatersleben.de/misa/ (accessed on 11 July 2024)) [41]. The minimum criteria for SSR identification were set as follows: mononucleotide repeats ≥ 10, dinucleotide repeats ≥ 6, and trinucleotide to hexanucleotide repeats ≥ 5. Primers were designed using Primer 3.0 online software [42].

### 4.5. Screening of Polymorphic SSR Primers

A total of 47 cpSSR and 145 EST-SSR primers were initially screened by genotyping four samples from different populations. The fluorescently labeled PCR analysis was performed following our previously established method [43]. The PCR amplification program was set as follows: 94 °C for 5 min; 30 cycles of 94 °C for 30 s, 58 °C for 30 s, and 72 °C for 1 min, followed by 13 cycles of 94 °C for 30 s, 53 °C for 30 s, and 72 °C for 1 min; and a final extension at 72 °C for 10 min. The PCR products were analyzed using an ABI 3730XL DNA Analyzer (Applied Biosystems, Waltham, MA, USA). After preliminary screening, 7 cpSSR and 14 EST-SSR polymorphic primers were selected for a further analysis of all samples.

### 4.6. Phylogenetic Analysis Based on Chloroplast Genome

The chloroplast genome sequences of seven species from the Zingiberales order were used for the phylogenetic analysis with the chloroplast genome of *O. chinensis*. These seven species include *Hellenia speciosa* (OK641589) from Costaceae, *Heliconia collinsiana* (NC_020362) from Heliconiaceae, *Ravenala madagascariensis* (NC_022927) from Strelitziaceae, *Musa balbisiana* (NC_028439) from Musaceae, *Zingiber officinale* (NA_044775) from Zingiberaceae, *Thalia dealbata* (NC_086578) from Marantaceae, and *Canna indica* (OR502631) from Cannaceae. A phylogenetic tree was generated utilizing 69 protein-coding genes (PCGs) common to eight complete chloroplast genomes within the order Zingiberales. Sequence alignment was conducted with MAFFT v7.475 [44]. The maximum-likelihood (ML) phylogenetic analysis was executed using IQ-Tree v1.6.10 [45] and subsequently visualized in MEGA7 [46].

### 4.7. Analysis of Genetic Diversity and Population Structure Based on SSR Markers

The Major Allele Frequency, number of alleles, gene diversity, and *PIC* index for each locus were calculated using Powermarker V3.25 [47]. Nei’s genetic distances among the 96 individuals and seven populations were also calculated, and an NJ tree was constructed based on genetic distances and visualized using MEGA7 [46]. PCoA and AMOVA were performed using GenAlex 6.5 [48]. The population genetic structure analysis was conducted using Structure 2.3.4 [49], with K values set from 1 to 10 using the admixture model. The optimal K value was determined using Structure Harvester [50] based on the Evanno method. CLUMPP v1.1.275 [51] was used to implement optimal alignment clustering. General linear regression was performed using built-in functions in R [52].

## 5. Conclusions

This study successfully assembled and analyzed the complete chloroplast genome of *O. chinensis* using high-throughput sequencing technology. The genome, with a total length of 134,407 bp, enabled the construction of an ML phylogenetic tree including representative species from eight families within Zingiberales, clarifying the phylogenetic position of *O. chinensis* and revealing its close relationship with *Ravenala madagascariensis* (Strelitziaceae). Seven cpSSRs and fourteen EST-SSRs were screened to evaluate the genetic diversity of 96 samples from seven wild populations, uncovering a complex pattern of genetic variation within and among populations. One cpSSR and eleven EST-SSR markers exhibited a high polymorphism (*PIC* > 0.5), indicating their high utility in assessing the genetic diversity of *O. chinensis* populations. The clustering analysis, PCoA, and population genetic structure analysis all indicated some genetic differentiation between G1 and G6 and the other five populations, but AMOVA results emphasized the greater contribution of within-population genetic variation to overall genetic diversity. In summary, the application of these new molecular markers will aid in monitoring population dynamics, evaluating conservation effectiveness, and exploring potential genetic resources, thereby promoting the recovery and long-term conservation of *O. chinensis* populations.

## Figures and Tables

**Table 1:** Gene composition in the chloroplast genome of *O. chinensis*.

Category of Genes	Group of Genes	Name of Genes
Genes for photosynthesis	Subunits of ATP synthase	*atpA*, *atpB*, *atpE*, *atpF*, *atpH*, *atpI*
	Subunits of photosystem II	*psbA*, *psbB*, *psbC*, *psbD* (3), *psbE*, *psbF*, *psbI*, *psbJ*, *psbK*, *psbL*, *psbM*, *psbN*, *psbT*, *psbZ*, *ycf3*
	Subunits of NADH dehydrogenase	*ndhA*, *ndhB*, *ndhC*, *ndhD*, *ndhE*, *ndhF*, *ndhG*, *ndhH*, *ndhI*, *ndhJ*, *ndhK*
	Subunits of cytochrome b/f complex	*petA*, *petB*, *petD*, *petG*, *petL*, *petN*
	Subunits of photosystem I	*psaA*, *psaB*, *psaI*, *psaJ*
	Subunit of rubisco	*rbcL*
Self-replication	Large subunit of ribosome	*rpl14*, *rpl16*, *rpl20*, *rpl22*, *rpl32*, *rpl33*, *rpl36*
	DNA-dependent RNA polymerase	*rpoA*, *rpoB*, *rpoC1*, *rpoC2*
	Small subunit of ribosome	*rps11*, *rps12*, *rps14*, *rps15*, *rps16*, *rps19*, *rps2*, *rps4*, *rps7*, *rps8*
	Transfer RNA	*trnQ-UUG*, *trnH-GUG* (2), *trnL-UAG*, *trnL-UAA*, *trnL-CAA*, *trnS-GCU*, *trnG-UCC* (2), *trnI-GAU*, *trnW-CCA*, *trnC-GCA*, *trnS-UGA*, *trnM-CAU* (3), *trnV-GAC*, *trnT-GGU*, *trnR-UCU*, *trnT-UGU*, *trnP-UGG*, *trnA-UGC*, *trnK-UUU*, *trnR-ACG*, *trnY-GUA*, *trnN-GUU*, *trnS-GGA* (2), *trnfM-CAU*, *trnV-UAC*, *trnF-GAA*, *trnE-UUC*, *trnD-GUC*
	Ribosomal RNA	*rrn23S*, *rrn4.5S*, *rrn16S*, *rrn5S*
Other genes	c-Type cytochrome synthesis gene	*ccsA*
	Envelop membrane protein	*cemA*
	Translational initiation factor	*infA*
	Maturase	*matK*
	Conserved open reading frames	*ycf4*

**Table 2:** Statistical values of seven cpSSRs and 14 EST-SSRs in 96 accessions across seven populations.

Item	Marker	Major Allele Frequency	*Na* *	*GD* *	*PIC* *
cpSSR	OccpSSR1	0.98	2	0.04	0.04
	OccpSSR8	0.89	2	0.2	0.18
	OccpSSR12	0.88	2	0.22	0.19
	OccpSSR19	0.76	2	0.36	0.30
	OccpSSR40	0.77	5	0.37	0.33
	OccpSSR44	0.72	5	0.46	0.43
	OccpSSR45	0.52	4	0.59	0.52
Mean		0.79	3.14	0.32	0.29
EST-SSR	OcESTSSR17	0.29	11	0.83	0.80
	OcESTSSR25	0.20	10	0.86	0.85
	OcESTSSR34	0.46	4	0.58	0.50
	OcESTSSR35	0.44	10	0.76	0.74
	OcESTSSR39	0.27	29	0.89	0.89
	OcESTSSR41	0.32	16	0.82	0.80
	OcESTSSR62	0.49	10	0.66	0.60
	OcESTSSR63	0.22	9	0.84	0.82
	OcESTSSR69	0.29	10	0.83	0.81
	OcESTSSR71	0.80	5	0.33	0.31
	OcESTSSR75	0.43	6	0.74	0.70
	OcESTSSR77	0.42	9	0.75	0.72
	OcESTSSR78	0.43	7	0.70	0.65
	OcESTSSR83	0.47	3	0.59	0.50
Mean		0.39	9.93	0.73	0.69

*: *Na*, number of alleles; *GD*, gene diversity; *PIC*, polymorphism information content.

**Table 3:** Summary of genetic statistics for seven populations of *O. chinensis*.

Populations	Sample Size	Major Allele Frequency	*Na* *	*GD* *	*PIC* *
G1	13	0.59	3.33	0.50	0.46
G2	18	0.67	3.43	0.46	0.42
G3	18	0.74	2.33	0.37	0.32
G4	16	0.78	2.76	0.31	0.28
G5	10	0.97	1.29	0.05	0.05
G6	11	0.90	1.62	0.14	0.12
G7	10	0.63	2.71	0.45	0.40

*: *Na*, number of alleles; *GD*, gene diversity; *PIC*, polymorphism information content.

**Table 4:** Summary of AMOVA result.

Source	df	SS	MS	Est. Var.	Variation
Among Pops	6	252.433	42.072	2.813	42%
Within Pops	89	344.556	3.871	3.871	58%
Total	95	596.990		6.684	100%

## Data Availability

The WGS data (PRJNA1170495) and RNA sequencing data (PRJNA1170418) were uploaded to NCBI.

## Supplementary Materials

The following supporting information can be downloaded at: https://www.mdpi.com/article/10.3390/ijms252011137/s1.

## References

[1] My S., Kalu M., Syazana A. (2019). *Orchidantha sarawakensis* sp. Nov. (Zingiberales: Lowiaceae), a new species endemic to east malaysia, borneo. J. Trop. For. Sci..

[2] Zou P., Cai X.A., Xu K., Cui C.J., Ye Y.S., Liao J.P. (2019). *Orchidantha crassinervia* sp. Nov. (Lowiaceae) from Guangxi, China. Nord. J. Bot..

[3] Leong-Skornickova J., Binh Nguy Q., Sida O. (2014). *Orchidantha virosa* Skornick. & Q.B.Nguyen, sp. Nov. (Lowiaceae), a new species endemic to northern vietnam. Adansonia.

[4] ăng Trân H., Leong-škorničková J. (2010). *Orchidantha stercorea* sp. Nov. (Lowiaceae) from vietnam. Nord. J. Bot..

[5] Cai L., Dao Z., Sun W. (2019). Discovery of a wild population of *Orchidantha yunnanensis* in south-east yunnan, China. Oryx.

[6] Johansen L.B. (2005). Phylogeny of *Orchidantha* (Lowiaceae) and the Zingiberales based on six dna regions. Syst. Bot..

[7] Niissalo M.A., Gardner E.M., Khew G.S., šída O., Poulsen A.D., Leong-škorničková J. (2022). Whence came these plants most foul? Phylogenomics and biogeography of Lowiaceae (Zingiberales). Front. Ecol. Evol..

[8] Kress W.J., Prince L.M., Hahn W.J., Zimmer E.A., Olmstead R., Olmstead R. (2001). Unraveling the evolutionary radiation of the families of the Zingiberales using morphological and molecular evidence. Syst. Biol..

[9] Barrett C.F., Specht C.D., Leebens-Mack J., Stevenson D.W., Zomlefer W.B., Davis J.I. (2014). Resolving ancient radiations: Can complete plastid gene sets elucidate deep relationships among the tropical gingers (Zingiberales)?. Ann. Bot..

[10] Sass C., Iles W.J.D., Barrett C.F., Smith S.Y., Specht C.D. (2016). Revisiting the Zingiberales: Using multiplexed exon capture to resolve ancient and recent phylogenetic splits in a charismatic plant lineage. PeerJ.

[11] Carlsen M.M., Fér T., Schmickl R., Leong-škorničková J., Newman M., Kress W.J. (2018). Resolving the rapid plant radiation of early diverging lineages in the tropical Zingiberales: Pushing the limits of genomic data. Mol. Phylogenet. Evol..

[12] Wen L., Zeng P., Zhang L., Huang W., Wang H., Chen G. (2016). Symbiosis theory-directed green synthesis of silver nanoparticles and their application in infected wound healing. Int. J. Nanomed..

[13] Luo Y., Chen W., Wen L., Zhou L., Kang X., Chen G. (2017). A new hexanedioic acid analogue from the endophytic fungus penicillium sp. Oc-4 of *Orchidantha chinensis*. Chem. Nat. Compd..

[14] Kirchoff B.K., Liu H., Liao J. (2020). Inflorescence and flower development in *Orchidantha chinensis* T. L. Wu (Lowiaceae; Zingiberales): Similarities to inflorescence structure in the strelitziaceae. Int. J. Plant Sci..

[15] Pedersen L.B., Johansen B. (2004). Anatomy of the unusual stigma in *Orchidantha* (Lowiaceae). Am. J. Bot..

[16] Song J., Liao J., Tang Y., Chen Z. (2004). Chromosome numbers in *Orchidantha* (Lowiaceae) and their biogeographic and systematic implications. Ann. Bot. Fenn..

[17] Zou P., Ye Y.S., Cai X.A., Liao J.P. (2014). Rediscovery and improved description of *Orchidantha insularis* (Lowiaceae), a rare species from hainan, china. Nord. J. Bot..

[18] Li R., Tang Y., Liao J. (2005). Study on genetic diversity of *Orchidantha chinensis* (Lowiaceae). J. Trop. Subtrop. Bot..

[19] Li R., Zou P., Tang Y., Liao J. (2008). Study on the population biology of endangered plant *Orchidanha*. J. Anhui Agric. Sci..

[20] Taheri S., Lee Abdullah T., Yusop M.R., Hanafi M.M., Sahebi M., Azizi P., Shamshiri R.R. (2018). Mining and development of novel SSR markers using next generation sequencing (NGS) data in plants. Molecules.

[21] Varshney R.K., Graner A., Sorrells M.E. (2005). Genic microsatellite markers in plants: Features and applications. Trends Biotechnol..

[22] Provan J., Powell W., Hollingsworth P.M. (2001). Chloroplast Microsatellites: New Tools for Studies in Plant Ecology and Evolution.

[23] Jakobsson M., Säll T., Lind-Hallden C., Hallden C. (2007). Evolution of chloroplast mononucleotide microsatellites in *Arabidopsis thaliana*. Theor. Appl. Genet..

[24] Schaal B.A., Hayworth D.A., Olsen K.M., Rauscher J.T., Smith W.A. (1998). Phylogeographic studies in plants: Problems and prospects. Mol. Ecol..

[25] Petit R.J., Duminil J., Fineschi S., Hampe A., Salvini D., Vendramin G.G. (2005). Comparative organization of chloroplast, mitochondrial and nuclear diversity in plant populations. Mol. Ecol..

[26] Ebert D., Peakall R. (2009). Chloroplast simple sequence repeats (cpSSRs): Technical resources and recommendations for expanding cpssr discovery and applications to a wide array of plant species. Mol. Ecol. Resour..

[27] Parthiban S., Govindaraj P., Senthilkumar S. (2018). Comparison of relative efficiency of genomic SSR and EST-SSR markers in estimating genetic diversity in sugarcane. 3 Biotech.

[28] Botstein D., White R.L., Skolnick M., Davis R.W. (1980). Construction of a genetic linkage map in man using restriction fragment length polymorphisms. Am. J. Hum. Genet..

[29] Hu L., Wang J., Wang X., Zhang D., Sun Y., Lu T., Shi W. (2024). Development of SSR markers and evaluation of genetic diversity of endangered plant *Saussurea involucrata*. Biomolecules.

[30] Szczecinska M., Sramko G., Wolosz K., Sawicki J. (2016). Genetic diversity and population structure of the rare and endangered plant species *Pulsatilla patens* (L.) Mill in east central europe. PLoS ONE.

[31] Sakai S., Inoue T. (1999). A new pollination system: Dung-beetle pollination discovered in *Orchidantha inouei* (Lowiaceae, Zingiberales) in sarawak, malaysia. Am. J. Bot..

[32] Kramer A.T., Havens K. (2009). Plant conservation genetics in a changing world. Trends Plant Sci..

[33] Volis S. (2016). How to conserve threatened chinese plant species with extremely small populations?. Plant Divers..

[34] Wauters L.A., Lens L. (1995). Effects of food availability and density on red squirrel (*Sciurus vulgaris*) reproduction. Ecology.

[35] Jin J., Yu W., Yang J., Song Y., Depamphilis C.W., Yi T., Li D. (2020). Getorganelle: A fast and versatile toolkit for accurate de novo assembly of organelle genomes. Genome Biol..

[36] Qu X., Moore M.J., Li D., Yi T. (2019). PGA: A software package for rapid, accurate, and flexible batch annotation of plastomes. Plant Methods.

[37] Wu M., Yan R., Xu X., Gou G., Dai Z. (2023). Characterization of the plastid genome of the vulnerable endemic *Indosasa lipoensis* and phylogenetic analysis. Diversity.

[38] Liu S., Ni Y., Li J., Zhang X., Yang H., Chen H., Liu C. (2023). CPGView: A package for visualizing detailed chloroplast genome structures. Mol. Ecol. Resour..

[39] Bolger A.M., Lohse M., Usadel B. (2014). Trimmomatic: A flexible trimmer for illumina sequence data. Bioinformatics.

[40] Grabherr M.G., Haas B.J., Yassour M., Levin J.Z., Thompson D.A., Amit I., Adiconis X., Fan L., Raychowdhury R., Zeng Q. (2011). Trinity: Reconstructing a full-length transcriptome without a genome from RNA-seq data. Nat. Biotechnol..

[41] Beier S., Thiel T., Münch T., Scholz U., Mascher M., Valencia A. (2017). Misa-web: A web server for microsatellite prediction. Bioinformatics.

[42] Koressaar T., Remm M. (2007). Enhancements and modifications of primer design program Primer3. Bioinformatics.

[43] Zhou Y., Ye Y., Zhu G., Xu Y., Tan J., Liu J. (2023). Diversity, classification, and est-ssr-based association analysis of caladium ornamental traits. Physiol. Plant..

[44] Katoh K., Standley D.M. (2013). Mafft multiple sequence alignment software version 7: Improvements in performance and usability. Mol. Biol. Evol..

[45] Nguyen L., Schmidt H.A., von Haeseler A., Minh B.Q. (2015). Iq-tree: A fast and effective stochastic algorithm for estimating maximum-likelihood phylogenies. Mol. Biol. Evol..

[46] Kumar S., Stecher G., Tamura K. (2016). Mega7: Molecular evolutionary genetics analysis version 7.0 for bigger datasets. Mol. Biol. Evol..

[47] Liu K., Muse S.V. (2005). Powermarker: An integrated analysis environment for genetic marker analysis. Bioinformatics.

[48] Peakall R., Smouse P.E. (2012). Genalex 6.5: Genetic analysis in excel. Population genetic software for teaching and research—An update. Bioinformatics.

[49] Pritchard J.K., Stephens M., Donnelly P. (2000). Inference of population structure using multilocus genotype data. Genetics.

[50] Earl D.A., Vonholdt B.M. (2012). Structure harvester: A website and program for visualizing structure output and implementing the evanno method. Conserv. Genet. Resour..

[51] Jakobsson M., Rosenberg N.A. (2007). Clumpp: A cluster matching and permutation program for dealing with label switching and multimodality in analysis of population structure. Bioinformatics.

[52] R Core Team (2023). R: A Language and Environment for Statistical Computing.

