# Supplementary material for: Assessing Genetic Diversity in Endangered Plant Orchidantha chinensis: Chloroplast Genome Assembly and Simple Sequence Repeat Marker-Based Evaluation
Source: Int J Mol Sci. 2024 Oct 17;25(20):11137. doi: 10.3390/ijms252011137 (PMC11508217; doi:10.3390/ijms252011137)
Supplement: Supplementary file 1 [file ijms-25-11137-s001.zip › Supplementary Figures.pdf]

Supplementary Figures

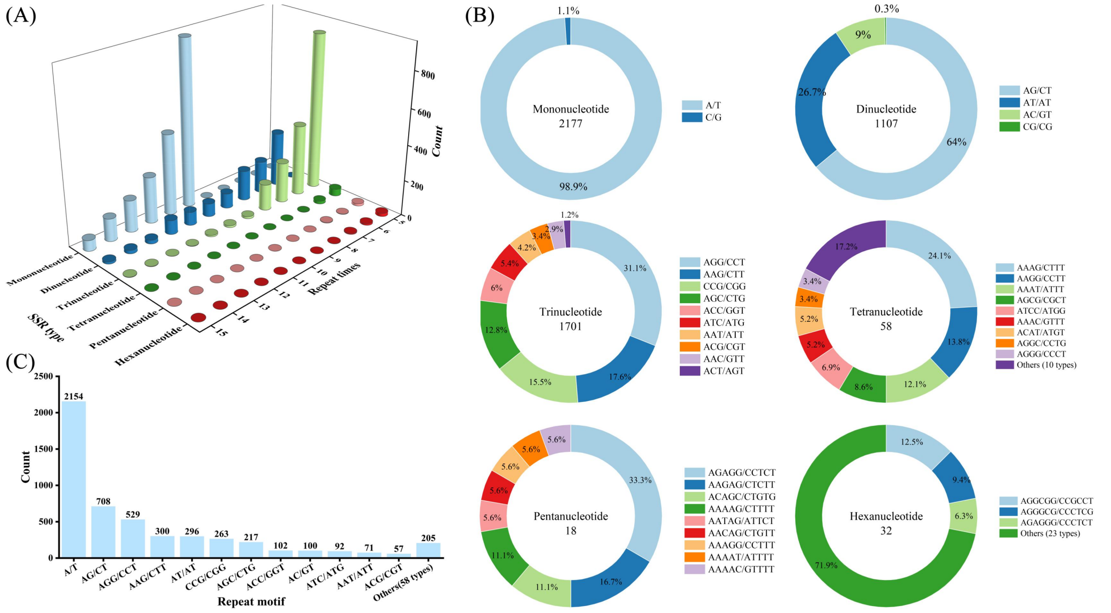

Figure S1. Quantitative characteristics of SSR in the transcriptome of *O. chinensis* leaves. (A) Distribution analysis of SSR with different repetition types and different repetition times. (B) Characteristics of repeat motif composition for different SSR repeat types. (C) Comparative analysis of the number of different SSR repeat motifs.

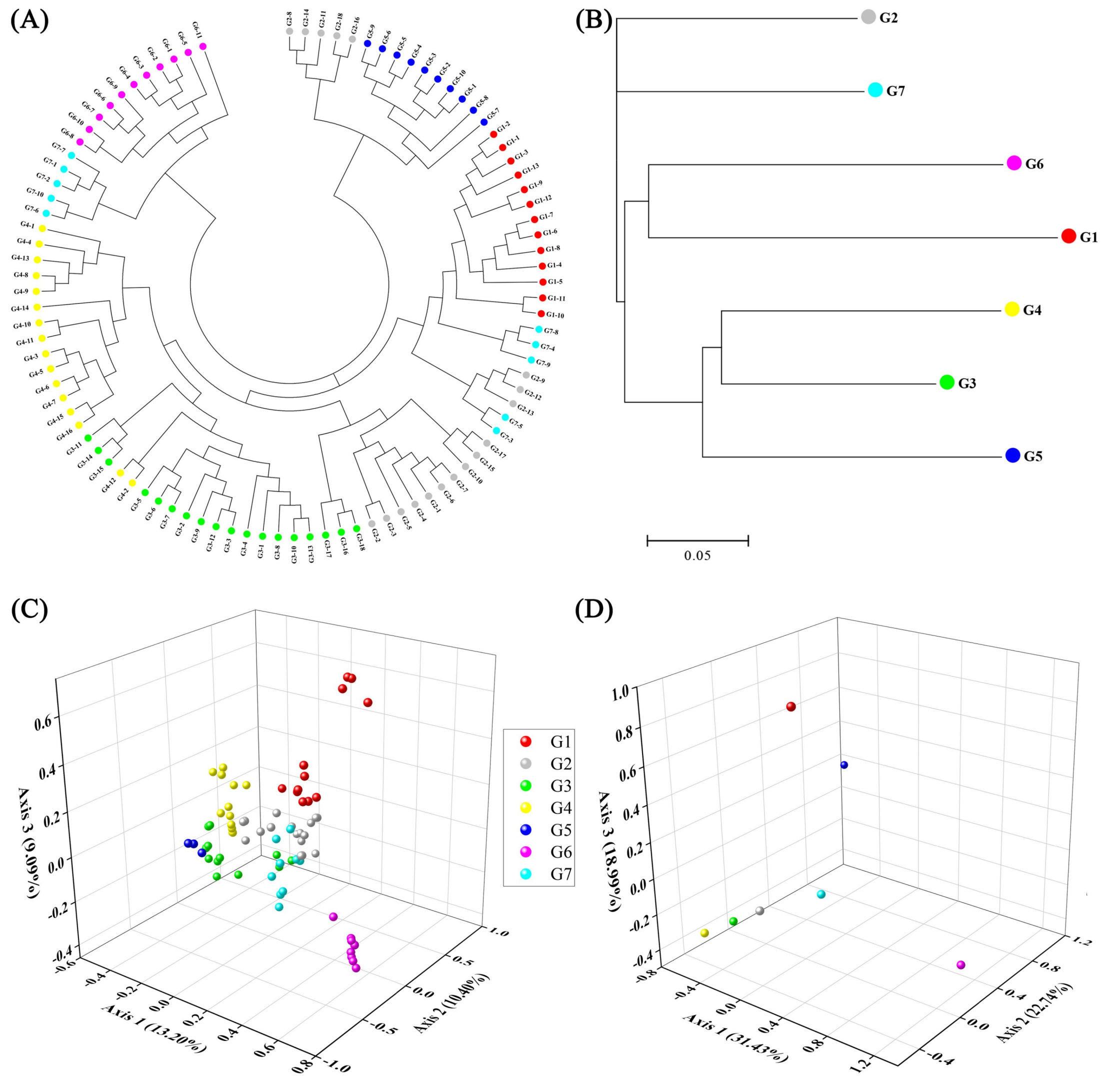

Figure S2. Genetic difference analysis of *O. chinensis* based on cpSSRs and EST-SSR. (A) NJ tree of 96 accessions. (B) NJ tree of seven populations. (C) Three D PCoA score plot of 96 accessions. (D) Three D PCoA score plot of seven populations.

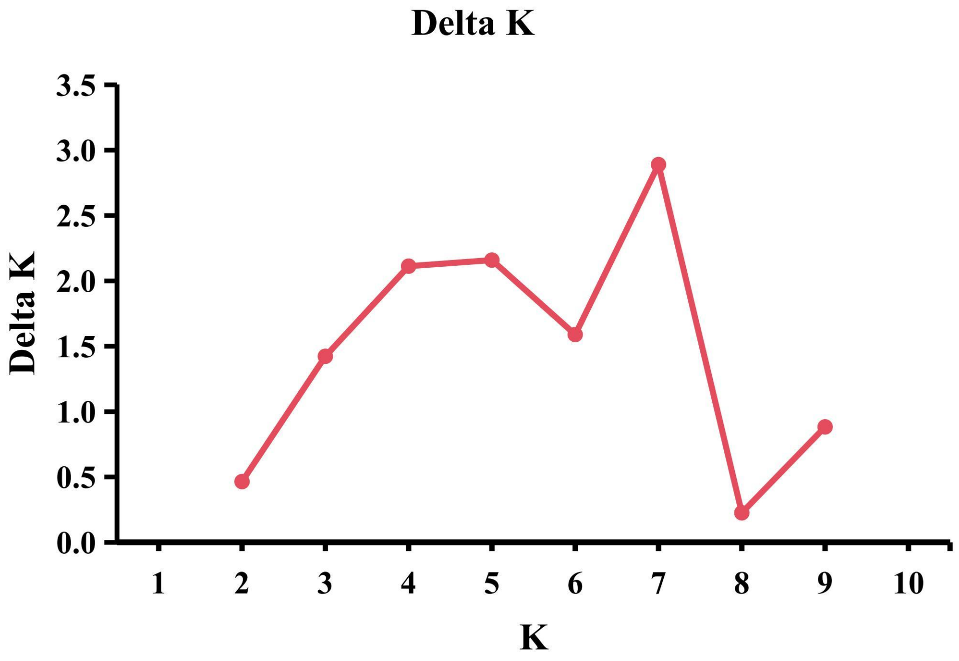

Figure S3. The value of delta K.

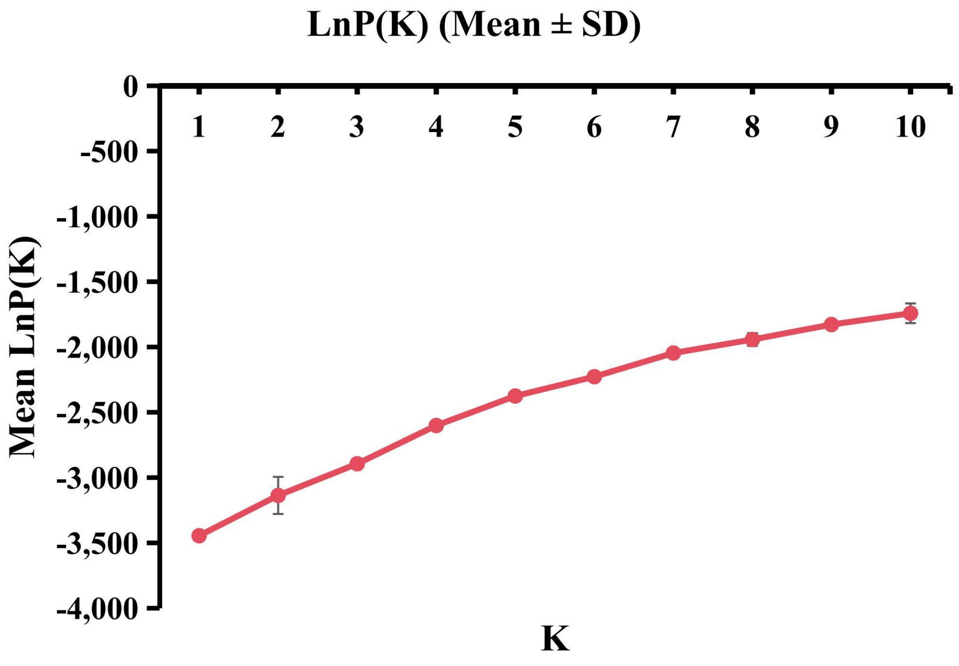

Figure S4. The value of LnP(K).
